# Effective transfer of chromosomes carrying leaf rust resistance genes from *Aegilops tauschii* Coss. into hexaploid triticale (*X Triticosecale* Witt.) using *Ae. tauschii* × *Secale cereale* amphiploid forms

**DOI:** 10.1007/s13353-014-0264-3

**Published:** 2014-12-14

**Authors:** Michał Kwiatek, Maciej Majka, Halina Wiśniewska, Barbara Apolinarska, Jolanta Belter

**Affiliations:** Institute of Plant Genetics, Polish Academy of Sciences, Strzeszyńska 34, 60-479 Poznań, Poland

**Keywords:** *Aegilops tauschii*, Fluorescence and genomic in situ hybridization, Gene sources, Meiosis, Mitosis, Resistance genes markers, Triticale

## Abstract

This paper shows the results of effective uses of a molecular cytogenetics toolbox and molecular marker to transfer leaf rust resistance genes from *Aegilops tauschii* × *Secale cereale* (DDRR, 2n = 4x = 28) amphiploid forms to triticale cv. Bogo (AABBRR, 2n = 6x = 42). The molecular markers of resistance genes and in situ hybridization analysis of mitotic metaphase of root meristems confirmed the stable inheritance of chromosome 3D segments carrying *Lr32* from the BC_2_F_2_ to the BC_2_F_5_ generation of (*Ae. tauschii* × *S. cereale*) × triticale hybrids. The chromosome pairing analysis during metaphase I of meiosis of BC_2_F_4_ and BC_2_F_5_ hybrids showed increasing regular bivalent formation of 3D chromosome pairs and decreasing number of univalents in subsequent generations. The results indicate that using amphiploid forms as a bridge between wild and cultivated forms can be a successful technology to transfer the D-genome chromatin carrying leaf rust resistance genes into triticale.

## Introduction

Triticale (*X Triticosecale* Witt.) was created using wide crosses to combine the valuable traits of wheat (*Triticum aestivum* L.) and rye (*Secale cereale* L.) (Aase 1930; O’Mara 1953; Jenkins 1969; Kiss and Videki 1971). At first, diseases did not appear to be a serious limitation to triticale production, probably because the areas of production of this crop were not conductive to cause serious shifts in pathogen virulence (Singh and Saari 1991). When triticale began to expand into new production areas, new hybrid pathotypes carrying virulence genes appeared (Arseniuk 1996). The most common diseases of triticale are caused by fungal pathogens, such as leaf rust (caused by *Puccinia triticina*) and powdery mildew (caused by *Blumeria graminis*) (Singh and Saari 1991 and Troch et al. 2014, respectively). One of the main breeding strategies for triticale improvement was to introduce D-genome chromosomes into 6x triticale (AABBRR). A number of efforts to produce triticale substitution lines were made. The simplest way to obtain D(A) or/and D(B) substitution lines is through octoploid (AABBDDRR) × tetraploid (AARR or BBRR) triticale crosses (Krowlow 1970; Lukaszewski et al. 1984; Apolinarska 1993). Another way to introduce D-chromosomes into triticale is using wild, diploid goatgrasses, which are ancestors carrying the D-genome, to create *Aegilops tauschii* Coss. (DD, 2n = 2x = 14) × *S. cereale* (RR, 2n = 2x = 14) hybrids (Fedak 1984; Cabrera et al. 1996). *Ae. tauschii* has approximately 23,000 protein coding genes and 1,200 NBS-LRR genes, which provides a large number of potential disease resistance loci (Jia et al. 2013). A number of resistance genes were transferred form *Ae. tauschii* to cultivated wheat. Resistance genes like *Lr21* (on chromosome 1DS), *Lr22a* (2DS), *Lr32* (3D), *Lr39* (2DS), *Lr40* (1DS), *Lr42* (1D), and *Lr43* (7DS) are the most essential for resistance durability (Rowland and Kerber 1974; Cox et al. 1994; Hussien et al. 1997; Raupp et al. 2001). However, the utilization of interspecific crosses between triticale and related species are arduous and prolonged because of genetic barriers of crossability controlled by *Kr* genes (Riley and Chapman 1967; Sitch et al. 1985; and Zheng et al. 1992, respectively). Moreover, the suppression of homologue chromosome pairing by the *Ph* genes may also interfere with the performance of distant crosses (Riley and Chapman 1958; Sears 1976; Lukaszewski and Kopecký 2010).

**Fig. 1:**
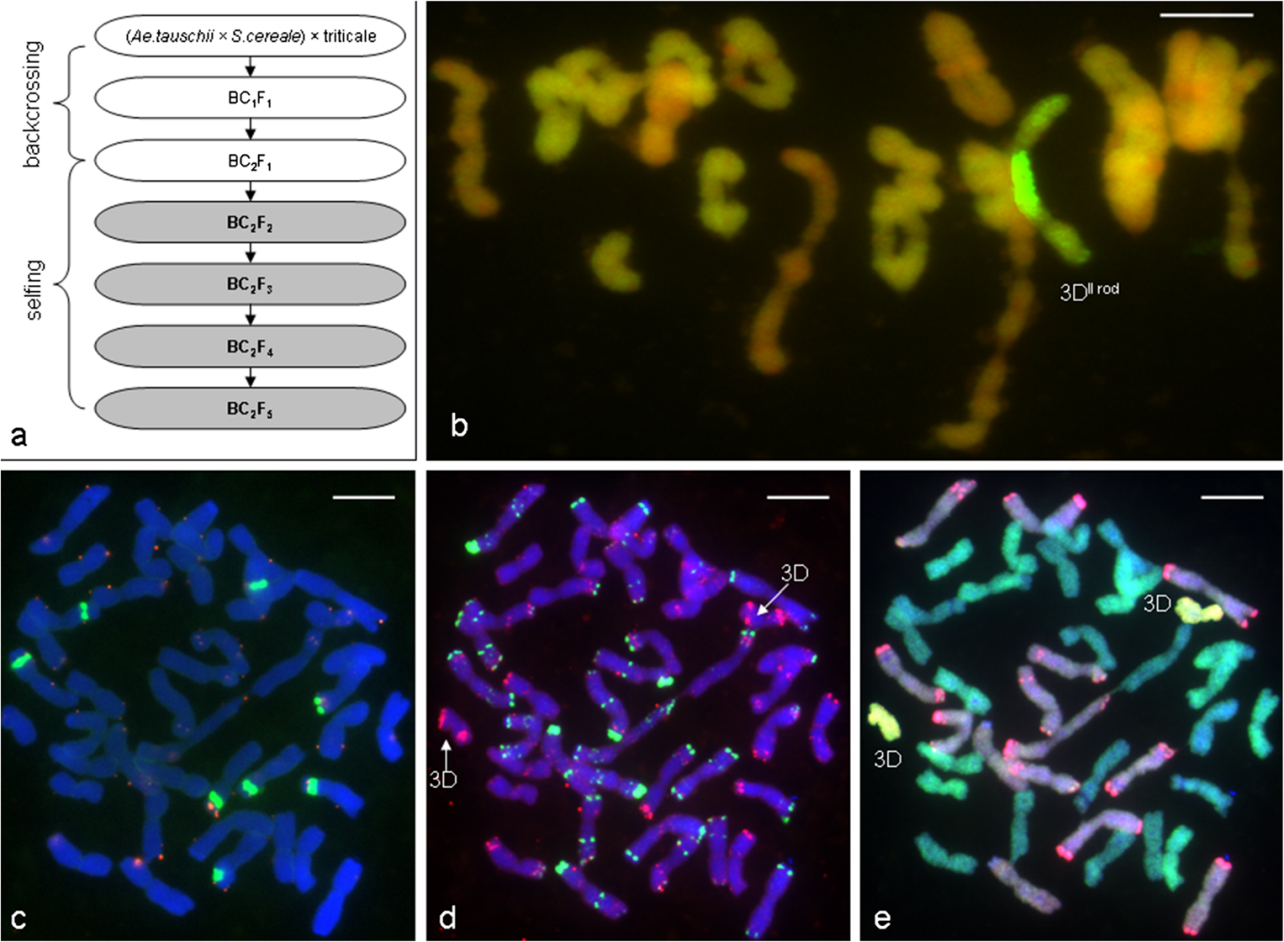
**a** The scheme of subsequent crosses between *Aegilops tauschii* × *Secale cereale* amphiploid forms and triticale cv. Bogo. Hybrids from the generations on distinguished fields were evaluated. **b** Genomic in situ hybridization (GISH) discrimination of *Ae. tauschii* chromosomes labeled using digoxigenin-11-dUTP (*green*) and unlabeled triticale chromosomes (*orange*) on meiotic metaphase I chromosome spread of pollen mother cells (PMCs) from the BC_2_F_5_ hybrid of (*Aegilops tauschii* × *Secale cereale*) × triticale, 20” + 1”3D(3B). **c** Fluorescence in situ hybridization (FISH) pattern showing the location of 5S rDNA (*red*) and 35S rDNA (*green*); **d** FISH pattern showing the location of pSc119.2 (*green*) and pAs1 (*red*) repetitive clones; and **e** GISH with a total genomic DNA from rye, R-genome, labeled with rhodamine (*red*); total genomic DNA from *Triticum monococcum*, A-genome, labeled with digoxigenin and detected by anti-digoxigenin conjugated with FITC (*green*/*yellow*); and total genomic DNA from *Aegilops tauschii*, D-genome, labeled with digoxigenin-11-dUTP and tetramethylrhodamine-5-dUTP (ratio 1:1) with blocking genomic DNA of *Aegilops speltoides*, B-genome (DAPI, *blue*) on mitotic, the same chromosome spread of the BC_2_F_5_ hybrid of (*Aegilops tauschii* × *Secale cereale*) × triticale

In this study, we have used the (*Aegilops tauschii* × *S. cereale*) × triticale hybrids, which were obtained by hybridization of the *Aegilops tauschii* × *S. cereale* amphiploids (DDRR, 2n = 4x = 28 chromosomes) with hexaploid triticale cv. Bogo as a paternal component to obtain the F_1_ generation of hybrids, which were, therefore, backcrossed with triticale ‘Bogo’ (pollinator) to obtain BC_1_F_1_ and BC_2_F_1_ hybrids, and further self-crossed to produce following generations (BC_2_F_2_ to BC_2_F_5_; Fig. 1a). The *Aegilops tauschii* × *S. cereale* amphiploids, produced using embryo rescue by Sulinowski and Wojciechowska of the Institute of Plant Genetics, Polish Academy of Sciences, Poznań, Poland (data unpublished), carried 28 chromosomes having *Lr22a* and *Lr39* (Kwiatek et al. 2012) and *Lr32* (data unpublished) markers connected with resistance to leaf rust. The aim of this study was: (1) to characterize the chromosome composition of the hybrids (BC_2_F_2_ to BC_2_F_5_) of *Ae. tauschii* × triticale hybrids; (2) to identify the D-genome DNA markers which are linked with leaf rust resistance in the hybrids; and (3) to evaluate the stability of D-genome chromosomes inheritance by the chromosome pairing analysis during MI (metaphase I) of meiosis of pollen mother cells (PMCs) of BC_2_F_4_ and BC_2_F_5_ hybrids.

The chromosome preparations for FISH/GISH (fluorescence/genomic in situ hybridization) analysis were made according to Hasterok et al. (2006). The MI (first metaphase) of PMCs meiosis preparation of chromosomes for the GISH experiment were made according to Schubert et al. (2001). The identification of particular chromosomes was performed by comparing the signal pattern of 5S rDNA, 25S rDNA, pSc119.2, and pAs1 probes according to a previous study (Kwiatek et al. 2013) and similar cytogenetic analysis (Cuadrado and Jouve 1994; Schneider et al. 2003, 2005; Wiśniewska et al. 2013). Genomic DNA from *Ae. tauschii* (D-genome), *Triticum monococcum* (A-genome), and *Secale cereale* (R-genome) was labeled by nick translation (using the Nick Translation Kit, Roche, Mannheim, Germany) with digoxigenin-11-dUTP (Roche) or tetramethyl-rhodamine-5-dUTP (Roche), depending on the visualization concept. The 5S rDNA probe was generated from the wheat clone pTa794 (Gerlach and Dyer 1980) by polymerase chain reaction (PCR) amplification and labeled by PCR with tetramethyl-rhodamine-5-dUTP using universal M13 sequencing primers. The 25S rDNA probe was made by nick translation of a 2.3-kb *Cla*I subclone of the 25S rDNA coding region of *Arabidopsis thaliana* (Unfried and Gruendler 1990) with digoxigenin-11-dUTP. The latter probe was used for the detection of 35S rDNA loci. The pSc119.2 repetitive DNA sequence was amplified and labeled by PCR with digoxigenin-11-dUTP by using universal M13 primers (Vrána et al. 2000). The probe pAs1 was amplified by PCR from the genomic DNA of *Ae. tauschii* and labeled with digoxigenin-11-dUTP, according to Nagaki et al. (1995). Digoxigenin was detected using anti-digoxigenin-rhodamine antibody (Roche). Chromosome and probe denaturation, hybridization, and post- hybridization washes were performed as described by Kwiatek et al. (2013). The identification of STS markers for *Lr22a* (*Xgwm296*), *Lr32* (*Xbarc135*), and *Lr39*(*Xgdm35*) were made according to Hiebert et al. (2007), Thomas et al. (2010), and Pestsova et al. (2000), respectively).

## Results and discussion

**Fig. 2:**
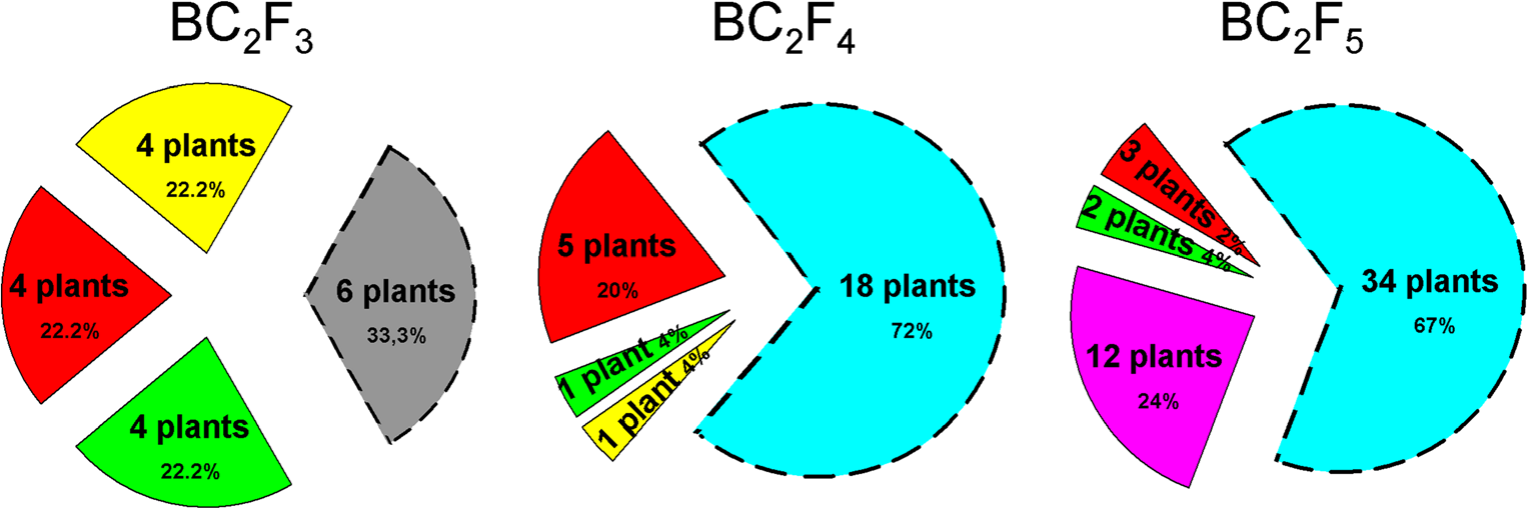
Number of plants in subsequent generations of respective chromosome set groups as follows: 20” + 1”3D + 1”2D (2n = 46; *gray*); 21” + 1’2D (2n = 43; *yellow*); 21” + 1’3D (2n = 43; *red*); 21” + 1”3D (2n = 44; *light blue*); 20” + 1’3D(3B) (2n = 42; *pink*); 21” + 0D (2n = 42; *green*). The *dotted line* indicates plants selected for self-crosses to obtain the next generation of hybrids

According to the literature, chromosome 2D of *Ae. tauschii* carries the *Lr22a* and *Lr39* markers (Hiebert et al. 2007 and Pestsova et al. 2000, respectively) and chromosome 3D possess the *Lr32* marker (Kerber 1987; Thomas et al. 2010) for leaf rust inheritance resistance genes. A sole plant exhibiting 46 chromosomes with two additional pairs of 2D and 3D chromosomes was chosen from BC_2_F_2_ hybrids for the molecular analysis. The products of *Lr22a* primers amplification resulted in 135 base pairs (bp) and 167 bp bands, which are characteristic for susceptible genotypes, according Hiebert et al. (2007). The PCR reaction using *Lr32* primers resulted in 261 bp and 273 bp products amplification, which indicate resistant genotypes. The *Lr39* marker was also identified in this plant using PCR, which resulted in a 185 bp product, appropriate for resistant genotypes. This plant was self-crossed to produce following generations (BC_2_F_3_ to BC_2_F_5_) (Fig 1a). The chromosome composition of hybrid plants was similar as generations advanced. The analysis of the progeny showed that six plants of the BC_2_F_3_ generation with addition of the pairs of 2D and 3D chromosomes also carried the *Lr32* and *Lr39* markers. Moreover, in four plants with additional 2D chromosome and four other plants with additional 3D chromosome, we have separately observed the amplification products for *Lr39* and *Lr32*, respectively. The BC_2_F_4_ plants were obtained from 21” + 1”3D + 1”2D genotypes (Table 1; Fig. 2). Fifteen plants with an additional pair of 3D chromosomes showed amplification products indicating heterozygosity (185 and 280 bp products; Pestsova et al. 2000) for the *Lr39* marker, although the FISH experiment presented a lack of 2D chromosomes in these plants. The same situation appeared in all plants with an additional pair of 3D chromosomes (34 plants) and in 12 plants with substitution pair 3D(3B) of the BC_2_F_5_ generation obtained from 21” + 1”3D genotypes (Table 1; Fig. 2).

**Table 1:** Results of the identification of STS markers for leaf rust resistance (*Lr*) in triticale with the introgression of D-genome chromatin from *Aegilops tauschii* × *S. cereale* amphiploids

Hybrid generation	Chromosome constitution of hybrids			Number of plants carrying *Lr* marker (number of heterozygous plants)		
	Number of plants	Appearance of D-genome chromosome(s)	2n	*Lr22a*	*Lr32*	*Lr39*
BC_2_F_2_	1	1”3D + 1”2D*	46	0	1	1
BC_2_F_3_	6	1”3D + 1”2D	46	0	6	6
	4	1’2D**	43	0	0	4
	4	1’3D	43	0	6	0
	4	0	42	0	0	0
BC_2_F_4_ from 21” + 1”2D + 1”3D	1	1’2D	43	0	0	1
	5	1’3D	43	0	5	0
	18	1”3D	44	0	18	0 (15)
	1	0	42	0	0	0
BC_2_F_5_ from 21” + 1”3D	3	1’3D	43	0	3	0
	34	1”3D	44	0	34	0 (34)
	12	1”3D(3B)***	42	0	12	0 (12)
	2	0	42	0	0	0

*Additional 2D chromosome pair and 3D chromosome pair

**Additional 2D chromosome

***Substitution pair 3D(3B)

It can be assumed that the high effectiveness of D-chromatin transfer from amphiploid forms (DDRR) into triticale (AABBRR) was caused by R-genome chromosomes pairing during prophase I of meiosis, which ensured the functional daughter cells formation. Moreover, the behavior of D-genome chromosomes studied during MI of meiosis of PMCs by GISH showed that the mean bivalent configurations increased when comparing BC_2_F_4_ and BC_2_F_5_ hybrids belonging to congruent plant groups divided with regard to the D-genome chromosome configuration (Table 2). The increasing mean number of the bivalents was similar to the study of Orlovskaya et al. (2007), which showed that the bivalent appearance in F_4_ hybrid plants of triticale × (*Aegilops* × *Triticum aestivum*; AABBUU and AABBS^sh^S^sh^) was substantially higher than in the F_1_ hybrids. The mean number of D-genome ring bivalents in the hybrids with an additional pair of 3D chromosomes (Table 2) was higher in BC_2_F_5_ plants (0.70) compared with BC_2_F_4_ plants (0.50). However, the mean number of D-genome rod bivalents decreased from 0.45 (BC_2_F_4_) to 0.25 (BC_2_F_5_). On the other hand, considering the same groups of plants, the mean number of D-genome univalents decreased from 0.18 to 0.08. According to the other studies concerning the meiotic analysis of *Aegilops* species with wheat and rye, the distant crosses should result in the intergenomic chromosome formation during meiosis. Molnár and Molnár-Láng (2010) observed the intergenomic rod and ring bivalents and trivalents between 2M, 3M, 3U, and 7M of *Ae. biuncialis* and wheat (Chinese Spring *ph1b*) chromosomes. In our study, we did not observe any intergenomic chromosome formation during meiosis. We suppose that triticale has the controlling system of homologue chromosome pairing that hampers the pairing of the chromosomes from different genomes. In wheat, homoeologous chromosome pairing and consequent recombination is suppressed by the function of the *Ph1* gene (Riley and Chapman 1958), located on chromosome 5B(5BL), and the *Ph2* gene on chromosome 3DS and 3AS (Mello-Sampayo 1971). The Chinese Spring *ph1b* (*CSph1b*) mutant genotype (Sears 1977), which lacks the *Ph1* locus, has been successfully used for the introgression of alien genetic material into the wheat genome by the induction of homoeologous pairing (Lukaszewski 2000). This is the reason for the intergenomic bivalent and trivalent appearance in the study of Molnár and Molnár-Láng (2010). In this work, the rDNA-FISH analysis and repetitive sequence FISH experiments showed that the pair of chromosomes 5B was present in all hybrids examined (Fig. 1c, d). This consideration led us to suppose that triticale cv. Bogo and the F_1_ to BC_2_F_5_ hybrids of (*Ae. tauschii* × *S. cereale*) × triticale cv. Bogo could carry the dominant allele of the *Ph1* gene. This assumption explained the appearance of 3D chromosomes in the following generations of hybrids, because only two homologue chromosomes can pair during meiosis and can be inherited from generation to generation.

**Table 2:** Chromosome configurations during metaphase I (MI) of meiosis of pollen mother cells (PMCs) in (*Aegilops tauschii* × *S. cereale*) × triticale hybrids

D-genome chromosome configuration in hybrid plants	Number of PMCs studied	Mean number of bivalents									Mean number of quadrivalents (RRRR)	Mean number of univalents		
		Mean number of bivalent configurations:								Total				
		Rods				Rings								
		Total	AB/AB	R/R	D/D	Total	AB/AB	R/R	D/D			A/B	R	D
BC_2_F_4_														
1’2D	40	4.50	4.15	0.35	0.00	16.30	9.65	6.65	0.00	20.80	0.00	2.10	0.00	0.95
1’3D	40	6.80	5.45	1.35	0.00	12.80	7.50	5.30	0.00	19.60	0.05	1.85	0.00	0.95
1”3D	80	4.65	3.45	0.75	0.45	14.75	8.95	5.30	0.50	19.40	0.15	3.25	0.00	0.18
BC_2_F_5_														
1’3D	40	7.56	6.33	1.23	0.00	12.96	7.18	5.78	0.00	20.52	0.00	2.00	0.00	0.98
1”3D	80	8.40	6.40	1.75	0.25	13.08	7.13	5.25	0.70	21.48	0.00	1.53	0.00	0.08

The stable inheritance of 3D chromosome pairs from the BC_2_F_2_ to BC_2_F_5_ generations of (*Ae. tauschii* × *S. cereale*) × triticale hybrids can be considered as a successful attempt of D-genome introgression into triticale, without using deletion or mutation lines of wheat or triticale with lack of the whole *Ph1* locus or *Ph1a* allele. The presence of the *Lr32* marker in the 49 BC_2_F_5_ plants carrying 3D chromosomes (Fig. 1 b, c, d, e) and the heterozygous character of the 46 BC_2_F_5_ plants considering the *Lr39* marker makes them useful components in breeding programs aimed at triticale improvement.
